# Divergent effects of itaconate isomers on *Coxiella burnetii* growth in macrophages and in axenic culture

**DOI:** 10.3389/fimmu.2024.1427457

**Published:** 2024-08-02

**Authors:** Md Nur A Alam Siddique, Fabian Kellermeier, Martha Ölke, Mingming Zhao, Konrad Büssow, Peter J. Oefner, Anja Lührmann, Katja Dettmer, Roland Lang

**Affiliations:** ^1^ Institute of Clinical Microbiology, Immunology and Hygiene, Universitätsklinikum Erlangen, Friedrich-Alexander-Universität Erlangen-Nürnberg, Erlangen, Germany; ^2^ Institute of Functional Genomics, University of Regensburg, Regensburg, Germany; ^3^ Department of Structure and Function of Proteins, Helmholtz Centre for Infection Research, Braunschweig, Germany

**Keywords:** ACOD1, IRG1, itaconate, mesaconate, citraconate, uptake, transport, infection

## Abstract

Aconitate decarboxylase-1 (ACOD1) is expressed by activated macrophages and generates itaconate that exerts anti-microbial and immunoregulatory effects. ACOD1-itaconate is essential for macrophage-mediated control of the intracellular pathogen *Coxiella (C.) burnetii*, which causes Q fever. Two isomers of itaconate, mesaconate and citraconate, have overlapping yet distinct activity on macrophage metabolism and inflammatory gene expression. Here, we found that all three isomers inhibited the growth of *C. burnetii* in axenic culture in ACCM-2 medium. However, only itaconate reduced *C. burnetii* replication efficiently in *Acod1^-/-^* macrophages. In contrast, addition of citraconate strongly increased *C. burnetii* replication in *Acod1^+/-^* macrophages, whereas mesaconate weakly enhanced bacterial burden in *Acod1^-/-^* macrophages. Analysis of intracellular isomers showed that exogenous citraconate and mesaconate inhibited the generation of itaconate by infected *Acod1^+/-^* macrophages. Uptake of added isomers into *Acod1^-/-^* macrophages was increased after infection for itaconate and mesaconate, but not for citraconate. Mesaconate, but not citraconate, competed with itaconate for uptake into macrophages. Taken together, inhibition of itaconate generation by macrophages and interference with the uptake of extracellular itaconate could be identified as potential mechanisms behind the divergent effects of citraconate and mesaconate on *C. burnetii* replication in macrophages or in axenic culture.

## Introduction

Expression of aconitate decarboxylase 1 (ACOD1), also known as immune response gene 1 (IRG1), is highly induced in mouse and human macrophages upon infection and TLR stimulation (1–4). ACOD1 localizes to mitochondria (1) and decarboxylates TCA cycle-derived cis-aconitate to generate the dicarboxylic acid itaconate (ITA) (5) that can be detected intracellularly in mouse macrophages at millimolar concentration (6–8). ITA has immunoregulatory activity and influences many cellular processes through activation of the anti-oxidant response to electrophilic stress, inhibition of enzymatic activities and by post-translational modification of signaling proteins (9–11). By attenuating the production of cytokines like IL-6, IL-12 and IL-1β in macrophages, ITA reduced lethality in the endotoxin model of sepsis (12–14) and may have potential as immunomodulatory treatment (15). On the other hand, ITA also has antimicrobial activity against several intracellular bacteria, including *Salmonella typhimurium* (6), *Mycobacterium tuberculosis* (5) and *M. avium* (16), *Legionella pneumophila* (4), *Francisella tularensis* (17), and *Brucella* spp (18).. In case of *F. tularensis*, ITA does not impair bacterial replication in axenic culture but acts indirectly on the intracellular bacterial replication by inhibiting the enzyme succinate dehydrogenase (SDH) that forms complex II of the mitochondrial respiratory chain (17). In contrast, ITA directly blocks replication of *M. tuberculosis*, *L. pneumophila* and *Brucella* spp. in axenic culture (4, 5, 18). For *M. tuberculosis* and *Brucella spp*, binding of ITA to isocitrate lyase and inhibition of the glyoxylate shunt appears to be the mechanism of its anti-bacterial effect (5, 18).

We recently showed that ACOD1 is required for macrophage-autonomous control of *C. burnetii* and that treatment with ITA inhibits the growth of *C. burnetii* in human and murine macrophages *in vitro* and in *Acod1^-/-^* mice *in vivo* (19). ITA directly inhibits replication of *C. burnetii* in cell-free, axenic culture in ACCM-2 medium (19, 20). The ITA concentration required to inhibit *C. burnetii* replication in macrophages and in axenic culture (< 5mM) can be easily achieved in activated macrophages (6). While these findings are compatible with the notion that ITA directly inhibits *C. burnetii* in infected macrophages, alterations of macrophage anti-microbial defense by ITA may also contribute.

Since the dicarboxylic acid ITA is a polar substance that does not pass passively through cell membranes, the cell-permeable esterified derivatives 4-octyl itaconate (4-OI) and dimethyl itaconate (DMI) have often been employed for treatment studies *in vitro* and *in vivo* (10). Both derivatives are more electrophilic than ITA and activate NRF2 more strongly than ITA (21). In addition, they are not converted into ITA intracellularly (21), although this has been challenged in the case of 4-OI (22). It is now clear that itaconate is released into the extracellular space by activated macrophages (23) in a process dependent on the ABCG2 transporter (24), and that exogenously added ITA is readily taken up by macrophages (19, 21, 25). However, it is still unknown whether uptake of ITA into macrophages involves specific transporter proteins.

**Figure 1:**
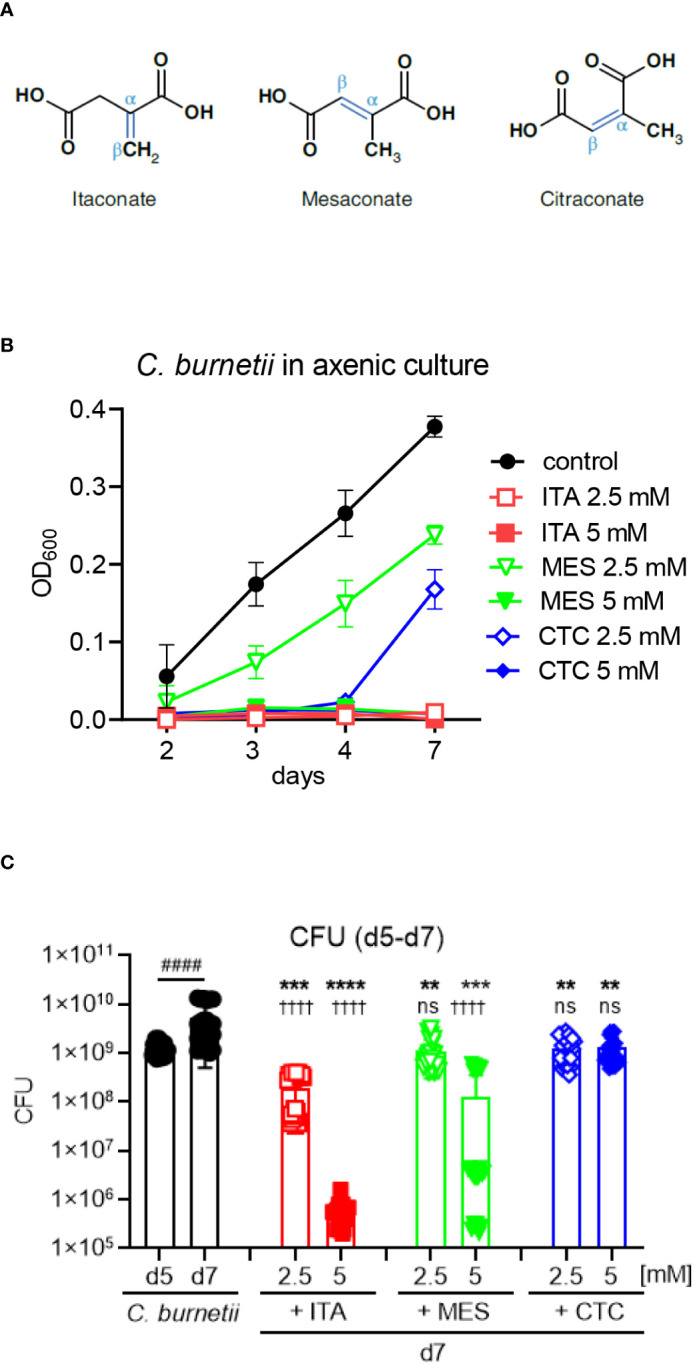
All isomers inhibit *C burnetii* replication in axenic culture, but ITA is more potent than MES and CTC. **(A)** Structures of the isomers ITA, MES and CTC. **(B)** ITA and its isomers were added to *C. burnetii* in ACCM-2 media on day 0. At the indicated time points, OD_600_ measurements were performed. Black closed symbols represent the untreated bacterial culture whereas red, green and blue color represents ITA, MES and CTC treatment, respectively. Concentration of 2.5 mM was indicated by open symbols and the closed symbols represent 5 mM of treatment. Data shown are mean and SD of 6 biological replicates pooled from three individual experiments. **(C)** *C. burnetii* growth inhibition by ITA and its isomers after delayed addition to the culture on day 5, followed by CFU determination on day 7. Data from four independent experiments, each with two biological and three technical replicates that were pooled together. Bars show mean with SD. Mann-Whitney test was performed individually between day 5 and day 7 mock treatment (^####^p<0.0001). To compare the effect of treatment versus mock, ANOVA was performed with Dunnett’s multiple comparison test. Treatment effects vs. day 7 mock: ****p<0.0001, ***p<0.001, **p<0.01. Treatment effects vs. day 5: ^††††^p<0.0001, *ns* not significant.

Recently, two isomers of ITA, mesaconate (MES) and citraconate (CTC) (**Figure 1A**) have been shown to be detectable in macrophages and to exert overlapping yet differential effects in macrophages compared to ITA. Generation of MES is ACOD1-dependent and inducible upon macrophage activation by LPS, albeit at less than one tenth of the concentration of ITA (25, 26). Similar to ITA, MES inhibits glycolysis and down-regulates expression of the pro-inflammatory cytokines IL-6 and IL-12, but it does not inhibit SDH (26). CTC can be detected by high-performance liquid chromatography – tandem mass spectrometry (HPLC-MS/MS) in human plasma and mouse lymph nodes and brain (27), but it was not detected in activated THP1 cells (25), a human monocyte/macrophage cell line, leaving the site of endogenous CTC production enigmatic. Exogenous CTC is the strongest electrophile and NRF2 activator in macrophages and reduced production of IL-6 and TNF, but did not inhibit SDH and mitochondrial respiration (25). Remarkably, CTC decreased the enzymatic activity of human and mouse ACOD1 by 90% and prevented accumulation of ITA and MES when added to LPS/IFNγ-activated THP1 cells (25).

Here, we explored the activity of the isomers MES and CTC, in comparison to ITA, on *C. burnetii* replication in axenic culture and in macrophages. All isomers inhibited *C. burnetii* growth in ACCM-2 medium, but only ITA restored control of bacterial replication in *Acod1^-/-^* macrophages, whereas CTC strongly increased *C. burnetii* load in *Acod1^+/-^* macrophages. All isomers reduced the production of IL-6 and TNF by infected macrophages. Gas chromatography-mass spectrometry (GC-MS) showed that only ITA was produced at a concentration above the lower limit of quantification (LLOQ) by *C. burnetii*-infected bone marrow-derived macrophages (BMM) and revealed that treatment with MES and CTC inhibited generation of ITA. Uptake of exogenous ITA and MES was upregulated in infected macrophages, and MES inhibited the uptake of ITA, suggesting competition for a shared uptake mechanism.

## Results

### All isomers inhibit *C. burnetii* replication in axenic culture, but ITA is more potent than MES and CTC

We have previously found that itaconate (ITA) inhibits the growth of *C. burnetii* in axenic culture. To determine whether the its isomers mesaconate (MES) and citraconate (CTC) (**Figure 1A**) share this activity, they were added at the beginning of axenic culture of *C. burnetii* and OD_600_ was measured to read out bacterial replication (**Figure 1B**). Confirming our previous results (19), ITA completely inhibited the growth of *C. burnetii* at 2.5 and 5 mM throughout the culture period of seven days. The isomers MES and CTC also abrogated bacterial growth completely when used at 5 mM. However, when used at 2.5 mM, CTC suppressed *C. burnetii* growth until day 4, and by 50% on day 7. Suppression of bacterial replication by MES was also incomplete at 2.5 mM with a significant 30-50% reduction in OD_600_ values at all time points.

We next asked whether ITA and its isomers can inhibit replication when added to established cultures on day 5 (**Figure 1C**). The number of viable bacteria was determined by CFU assays before (day 5) and two days after adding ITA, MES or CTC or medium control (day 7). *C. burnetii* continued to grow between day 5 and day 7 in medium. All compounds significantly reduced the CFU counts at 2.5 and 5 mM, when compared to medium only on day 7. However, ITA displayed the strongest, dose-dependent activity, with a clear bactericidal effect for the higher concentration that reduced CFU count by a factor of 1000 compared to the starting number on day 5, again confirming our previous results for ITA (19). MES and CTC reduced CFU numbers less efficiently than ITA at both concentrations, with MES showing an intermediate bactericidal activity at the 5 mM concentration, whereas CTC at both concentrations was bacteriostatic but did not reduce the CFU below the starting numbers on day 5 (**Figure 1C**). Taking together we noticed an inhibitory effect of ITA, MES and CTC on the replication of *C. burnetii* in axenic culture, with ITA as the most potent isomer.

### Only ITA inhibits, whereas CTC enhances *C. burnetii* replication in macrophages

**Figure 2:**
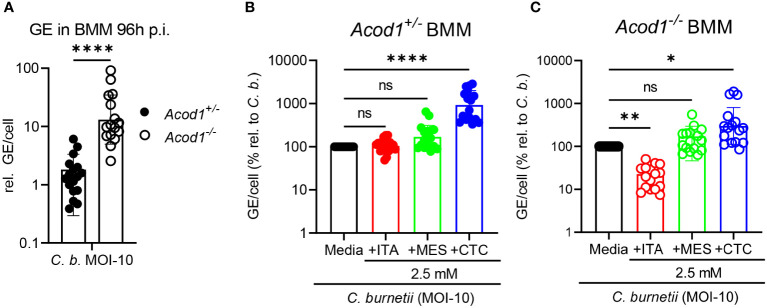
Only ITA inhibits, whereas CTC enhances *C. burnetii* replication in macrophages. *Acod1^+/-^* and *Acod1^-/-^* BMM were infected with *C. burnetii* at MOI-10 for 4 hours, followed by a washing step to remove extracellular bacteria. **(A)** *C. burnetii* genome equivalents (GE) per cell were measured by qPCR at 96 hours post infection from macrophage lysates. Average GE values from duplicate wells at 96 hours infection were normalized to the mean GE of all *Acod1^+/-^* control samples of 4h time point (initial uptake by BMM). **(B, C)** relative GE for BMM infected and treated for 96 hours with isomers was normalized to the infection alone condition (set to 100%). **(B)** *Acod1^+/-^*, **(C)** *Acod1^-/-^*. Each dot represents BMM from one mouse (average of 2 biological replicates, n= 17 mice per genotype) and data were pooled together from 6 individual experiments. Bars show geometric mean with geometric SD. Mann-Whitney test **(A)** and Friedman test (paired, non-parametric) **(B, C)** were performed between the indicated conditions. *p<0.05, **p<0.01, ****p<0.0001.

Next, we investigated the effect of ITA and its isomers in controlling intracellular *C. burnetii* replication in bone marrow-derived macrophages (BMM). BMM were infected for four hours with *C. burnetii* at MOI 10, followed by washing away the extracellular bacteria and addition of ITA, MES or CTC. BMM deficient in ACOD1, the inducible mitochondrial enzyme responsible for ITA production, cannot restrict NMII replication and contain a >10-fold higher bacterial burden compared to *Acod1^+/-^* BMM at 96 hours post infection (**Figure 2A**), as shown before (19). The effect of supplementation of ITA or its isomers at a concentration of 2.5 mM on intracellular *C. burnetii* bacterial load was determined for *Acod1^+/-^* (**Figure 2B**) and *Acod1^-/-^* BMM (**Figure 2C**). In *Acod1^+/-^* BMM no noticeable change was detected in controlling *C. burnetii* replication after ITA and MES treatment, whereas CTC caused a significant increase in *C. burnetii* genome equivalents (GE) (**Figure 2B**). In contrast, in *Acod1^-/-^* BMM that are unable to generate endogenous ITA, the supplementation of ITA restored the control of *C. burnetii*, confirming our previous results (19), whereas MES had no effect, and CTC caused a moderate increase in bacterial load (**Figure 2C**).

Thus, we observed very distinct effects of the three isomers on *C. burnetii* replication in macrophages: (1) ITA added to infected BMM did not reduce bacterial load in *Acod1^+/-^* BMM but restored control of replication in *Acod1^-/-^* BMM, (2) MES had no significant impact on *C. burnetii* load in both genotypes, and (3) CTC strongly enhanced *C. burnetii* load in *Acod1^+/-^* BMM and to some extent also in *Acod1^-/-^* BMM. The results for MES and CTC are in striking contrast to the direct inhibitory effect of these isomers on *C. burnetii* replication in axenic culture (**Figure 1**).

### Similar effects of isomers on cytokine production by *C. burnetii*-infected macrophages

**Figure 3:**
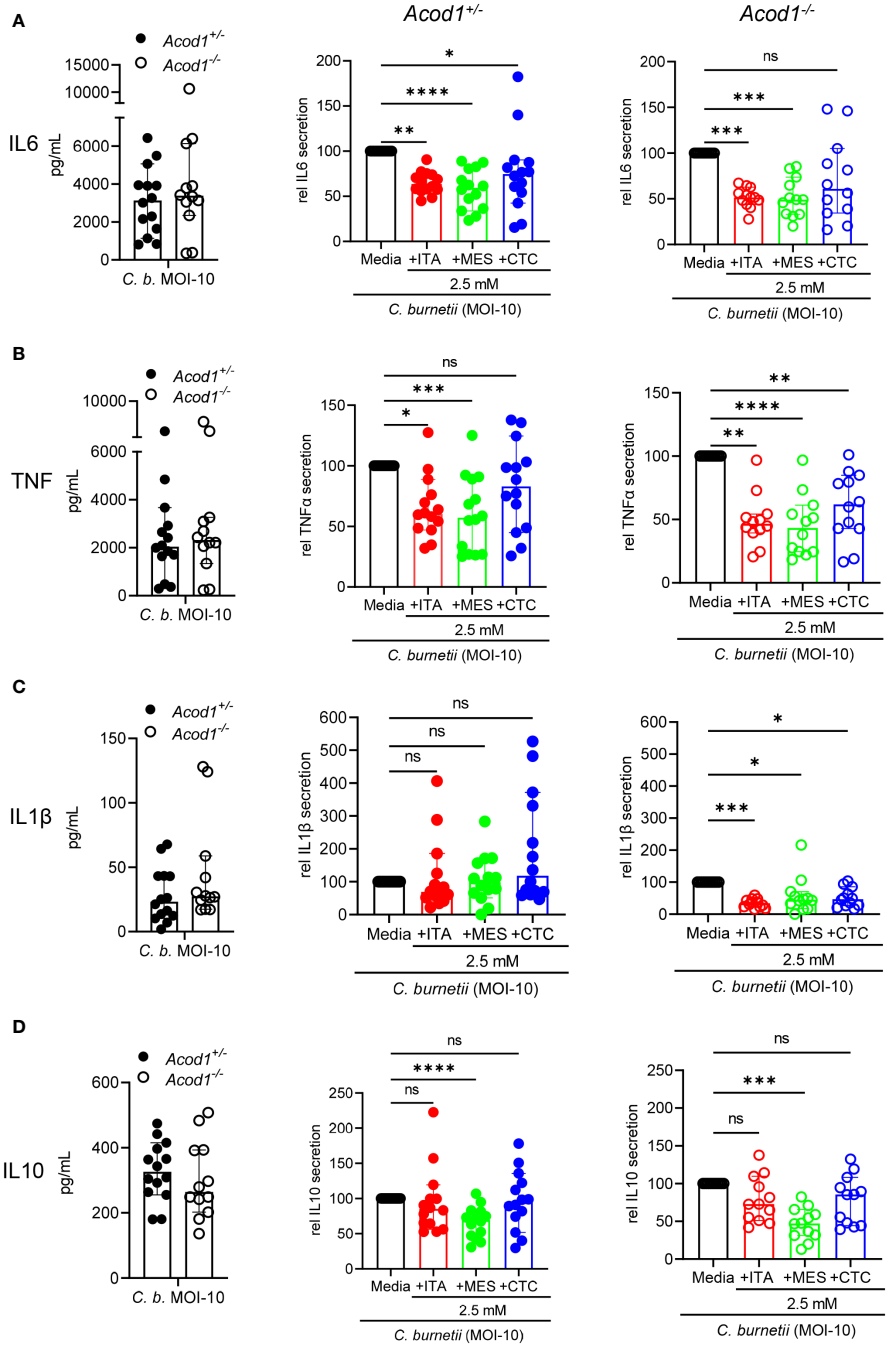
Similar effects of isomers on cytokine production by macrophages infected with *C. burnetii*. Production of the cytokines IL-6 **(A)**, TNF **(B)**, IL-1β **(C)** and IL-10 **(D)** was measured by ELISA from the cell culture supernatants of *C. burnetii* infected *Acod1^+/-^* and *Acod1^-/-^* BMM. 4h after infection extracellular bacteria were removed by a washing step followed by the supplementation of different treatment and/or media alone considering this time point as 0 hours. After 24 hours cell culture supernatant was collected, and cytokines were measured. For each of the analyzed cytokines, the left panel shows the concentration from infection alone comparing *Acod1^+/-^* and *Acod1^-/-^* BMM. The middle and right panels depict the comparison between untreated vs. treated separately for *Acod1^+/-^* and *Acod1^-/-^* BMM, respectively, setting the cytokine concentration from infection alone as 100% separately for *Acod1^+/-^* and *Acod1^-/-^* BMM. Each dot represents BMM from one mouse (average of 2 biological replicates) and data were pooled together from 6 individual experiments (n= 12-14 mice per genotype). Bars show median with 95% CI. Friedman test (paired, non-parametric) was performed between the indicated conditions. *p<0.05, **p<0.01, ***p<0.001, ****p<0.0001 **(A-D)**.

To explore whether the divergent effects of ITA isomers on *C. burnetii* replication in BMM and in axenic culture are associated with differences in macrophage activation, we next investigated their effect on cytokine production. As ITA is an immunomodulatory agent, we measured the pro-inflammatory cytokines TNF, IL-6, and IL-1β, and the anti-inflammatory cytokine IL-10 in cell culture supernatants 24 hours after NMII infection of *Acod1^+/-^* and *Acod1^-/-^* BMM (**Figure 3**). All four cytokines were produced by infected *Acod1^+/-^* and *Acod1^-/-^* BMM at comparable levels (left panels, **Figure 3**). However, the addition of ITA and its isomers did affect cytokine production in both BMM genotypes. ITA and MES reduced the production of IL-6 and TNF, while CTC did so for IL-6 in *Acod1^+/-^* and for TNF in *Acod1^-/-^* BMM (**Figures 3A, B**). The secretion of IL-1β was reduced by all three isomers, but only in *Acod1^-/-^* BMM (**Figure 3C**), which showed a trend for higher IL-1β secretion compared to *Acod1^+/-^* BMM. IL-10 production was reduced significantly by MES in both BMM genotypes (**Figure 3D**). Taken together, ITA and its isomers MES and CTC have an overall similar immunomodulatory effect on production of the cytokines IL6, TNFα, IL1β and IL10 in response to infection with *C. burnetii*. Thus, the differential effects of ITA, MES and CTC on *C. burnetii* control in BMM are unlikely to be due to differences in modulation of the investigated cytokines. Instead, other mechanisms must underly the observed specific inhibition of intracellular *C. burnetii* replication by ITA but not MES or CTC.

### Production of endogenous ITA and uptake of isomers in macrophages

**Figure 4:**
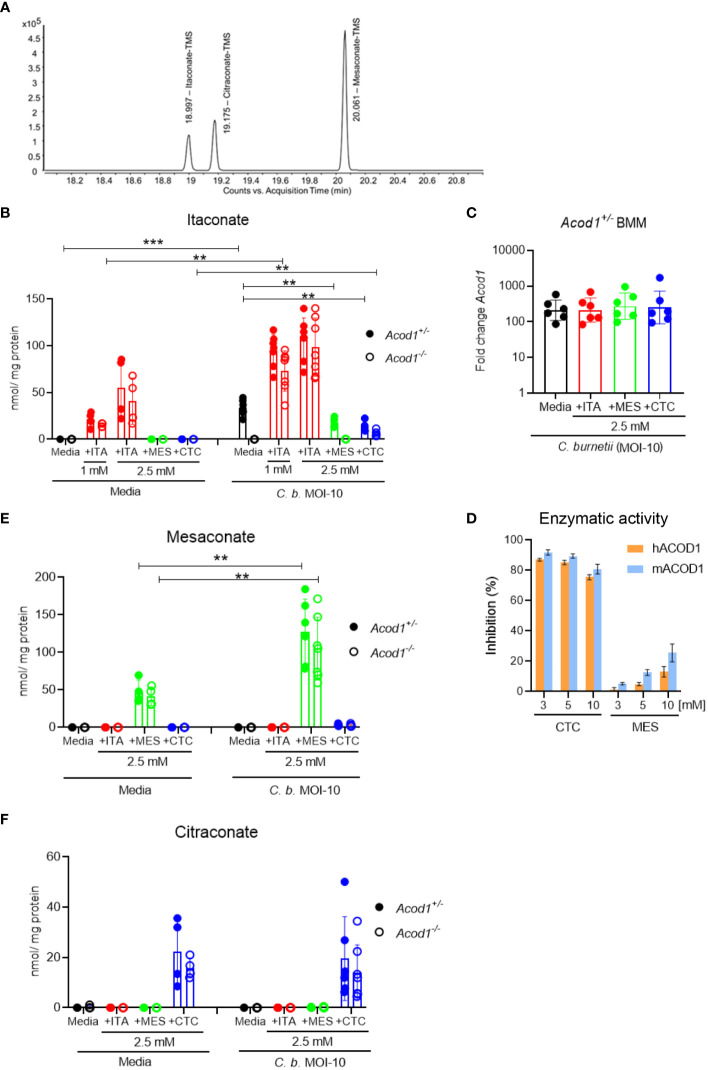
Production of endogenous ITA and uptake of isomers in macrophages. GC-MS analysis of intracellular ITA and its isomers in resting as well as *C. burnetii*-infected BMM. BMM were infected for 4 hours, followed by a washing step, then exogenous ITA, MES or CTC were added at the indicated concentrations. BMM was harvested after 24 hours of treatment. **(A)** Extracted ion chromatogram of m/z = 259.1 of a standard mixture containing ITA, MES and CTC. **(B, E, F)** Quantification of intracellular concentration of ITA **(B)**, MES **(E)** and CTC **(F)**, normalized to protein content. Each dot represents BMM from one mouse (average of 3 biological replicates) and data shown here are pooled from 2 individual experiments for resting BMM (n= 5-6 mice per genotype) and 3 individual experiments for infected BMM (n= 7-8 mice per genotype). Bars show mean with SD. Mann-Whitney test was performed separately between the indicated conditions **(B, E, F)**. **p<0.01, ***p<0.001. **(C)** *Acod1* mRNA expression levels analyzed by qRT-PCR in *Acod1^+/-^* BMM 24 hours after infection with *C. burnetii*. The different isomers were added at 2.5 mM to the BMM after removal of extracellular *C burnetii*. Data were calibrated to non-infected macrophages. Each dot represents one BMM from one donor mouse (n=6). **(D)** Test for inhibition of ACOD1 enzymes by MES *in vitro*. MES could not inhibit hACOD1 (orange) or mACOD1 (blue) efficiently in comparison to the known inhibitor CTC. *In vitro* production of ITA by purified ACOD1 proteins was measured by HPLC. Inhibition was calculated as the ratio of produced itaconate in the presence and absence of the compounds. Shown are mean and SD from three replicates.

To investigate the endogenous production and the uptake of exogenously supplemented ITA, MES and CTC, we performed gas chromatography-mass spectrometry (GC-MS)-based analysis in resting as well as in *C. burnetii*-infected mouse BMM (**Figure 4**). Peaks for the three isomers could be reliably separated by GC-MS (**Figure 4A**). Endogenous itaconate was not detectable above the LLOQ in resting BMM, whereas upon *C. burnetii* infection we noticed a strong increase in intracellular ITA levels (about 35 pmol/µg protein) in *Acod1^+/-^* but not in *Acod1^-/-^* BMM (**Figure 4B**), consistent with ACOD1 being the critical enzyme for itaconate production. The use of *Acod1^-/-^* BMM thus allowed us to distinguish between endogenously produced ITA and ITA taken up from cell culture medium, or being converted from exogenous MES or CTC after uptake. Following supplementation of 1 mM or 2.5 mM ITA, we observed dose-dependent increases in intracellular ITA in resting and infected BMM that exceeded the level of endogenously produced ITA in infected *Acod1^+/-^* BMM (**Figure 4B**). Of note, the intracellular concentration of ITA was several-fold higher in infected compared to resting *Acod1^-/-^* BMM, indicating that uptake of ITA was increased in activated macrophages (**Figure 4B**). We did not observe any conversion of exogenously added MES or CTC into ITA in resting BMM, whereas in infected BMM a minimal conversion of CTC into ITA was noticed in *Acod1^-/-^* BMM (**Figure 4B**). Remarkably, addition of CTC as well as of MES reduced production of ITA in infected *Acod1^+/-^* BMM (**Figure 4B**). This effect was expected for CTC that inhibits enzymatic activity of ACOD1, while such an effect has not been reported for MES (25). We considered the possibility that MES may down-regulate the expression of ACOD1. However, qRT-PCR measurement of *Acod1* mRNA in BMM infected for 24 hours with *C. burnetii* did not show any effect of ITA, MES or CTC (**Figure 4C**). Therefore, the impact of MES on the enzymatic activity of recombinant ACOD1 enzyme was assessed in an *in vitro* assay in comparison to CTC. While CTC, as described before, exerted inhibition of ITA production (25), addition of MES to murine or human ACOD1 at different concentrations had no impact on its activity (**Figure 4D**).

Next, we analyzed the intracellular levels of MES (**Figure 4E**) and CTC (**Figure 4F**) in resting and infected BMM. Unlike ITA, no endogenously generated MES or CTC was detectable by GC-MS above the lower limit of quantification in resting or infected BMM, which is at variance with recent reports (25, 26). However, LLOQs reported for the method (Lit: Metabolites 2021, 11, 270 (27). used by Chen et al. (24) are lower than ours. In contrast, exogenously supplemented MES and CTC were readily detected intracellularly by our GC-MS method (**Figures 4E, F**). Supplemented MES led to higher intracellular levels in *C. burnetii*-infected compared to resting BMM (**Figure 4E**), similar to what had been observed for ITA (**Figure 4B**). In contrast, addition of CTC yielded comparable intracellular levels in resting and infected BMM (**Figure 4F**). While the intracellular levels of ITA and MES were comparable after exogenous supplementation, the intracellular CTC concentration was considerably lower (ca. 2-fold lower in resting and at least 5-fold lower in *C. burnetii*-infected BMM), although all isomers were added at the same concentration of 2.5 mM (**Figures 4B, E, F**).

### Impact of MES and CTC on the uptake and anti-Coxiella activity of ITA in BMM

**Figure 5:**
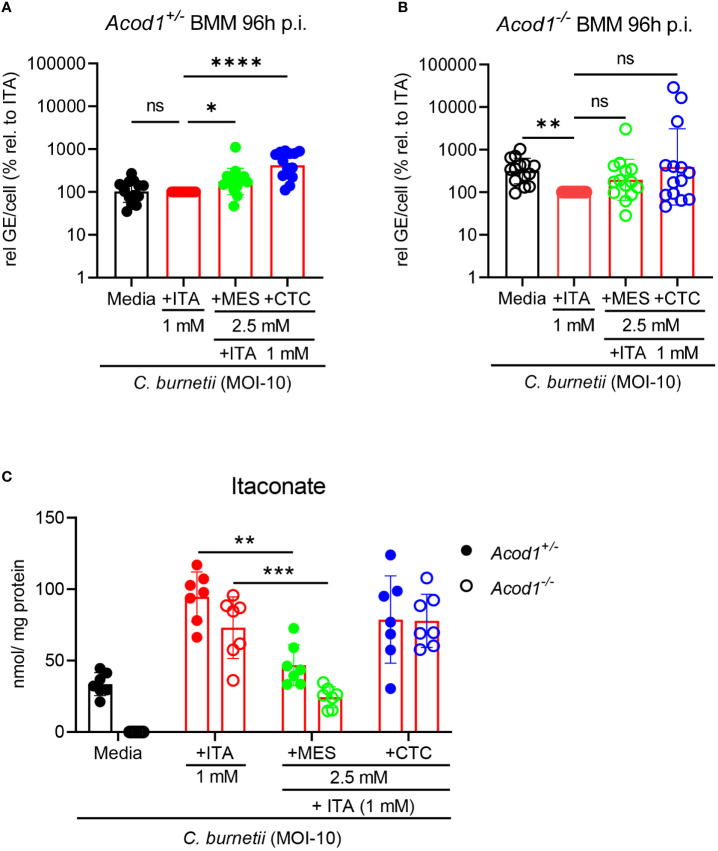
Impact of MES and CTC on uptake of ITA and its anti-*Coxiella* activity in BMM. **(A, B)** The impact of combined treatment, ITA together with MES or CTC supplementation in controlling *C burnetii* replication was investigated. Average GE values from duplicate wells at 96 hours post infection were normalized to the mean GE of all *Acod1^+/-^* control samples at 4 hours (initial bacterial uptake by the BMM). % relative to ITA was calibrated to the GE of *Coxiella burnetii* infected ITA treatment at 96 hours set to 100% for *Acod1^+/-^* and *Acod1^-/-^* BMM separately in each experiment. Each dot represents BMM from one mouse (average of 2 biological replicates, n=14 mice per genotype) and data were pooled together from 5 individual experiments. Bars show geometric mean with geometric SD. Friedman test (paired, non-parametric) **(A, B)** were performed between the indicated conditions. *p<0.05, **p<0.01, ****p<0.0001. **(C)** Intracellular ITA in *Coxiella burnetii* infected *Acod1^+/-^* and *Acod1^-/-^* BMM after treatment with ITA alone or combined treatment with ITA + MES/CTC was measured after 24 hours by GC-MS. BMM were infected for 4 hours, followed by a washing step. Then, where indicated, exogenous ITA, MES and CTC were added at the indicated concentrations. BMM were harvested after 24h treatment. Each dot represents BMM from one mouse (average of 3 biological replicates) and data shown here from 3 individual experiments (n=7 mice per genotype). Bars show mean with SD. Mann-Whitney test was performed separately between the indicated conditions. **p<0.01, ***p<0.001.

Finally, as only ITA, but not MES or CTC, reduced the *C. burnetii* load in infected BMM, we asked whether combined supplementation with ITA together with MES or CTC has an impact on control of intracellular bacteria by *Acod1^+/-^* and *Acod1^-/-^* BMM. Infected BMM were treated with itaconate (1 mM) alone or in combination with the isomers MES or CTC (2.5 mM), followed by analysis of *C. burnetii* burden after 96 hours (**Figures 5A, B**) and measurement of intracellular ITA by GC-MS after 24 hours (**Figure 5C**).

As observed with single isomer treatment (**Figure 2B**), CTC significantly increased *C. burnetii* burden in *Acod1^+/-^* BMM also when ITA was supplemented (**Figure 5A**). MES had a similar but weaker effect on *C. burnetii* levels in *Acod1^+/-^* BMM (**Figure 5A**). Both MES and CTC did not cause significant changes in bacterial load in ITA-treated *Acod1^-/-^* BMM, although there was a trend for higher *C. burnetii* GE (**Figure 5B**).

GC-MS analysis revealed that the addition of MES significantly reduced the intracellular level of ITA by 50% in *Acod1^+/-^* and by 65% in *Acod1^-/-^* BMM (**Figure 5C**). Thus, in addition to the observed inhibition of endogenous ITA production when MES was added alone (**Figure 4B**), MES blocks the uptake of ITA into infected BMM, which may contribute to the increase in bacterial burden (**Figures 5A, B**). In contrast, CTC supplementation did not alter the uptake of ITA in *Acod1^-/-^* BMM and only slightly reduced intracellular ITA concentration in *Acod1^+/-^* BMM (**Figure 5C**), which can be explained by its inhibition of endogenous ITA production (**Figure 4B**). Together, the mass spectrometry data showed that MES, but not CTC, interfered with the uptake of ITA into BMM. Thus, the isomers MES and CTC can interfere with ITA-mediated control of *C. burnetii* in BMM *via* different mechanisms, inhibition of ACOD1 enzymatic activity or blockade of uptake of extracellular ITA.

## Discussion

In this comparative analysis of the impact of ITA and its isomers on *C. burnetii*, we found a striking difference in the effects of MES and CTC on bacterial growth in axenic culture and intracellularly in macrophages. The discrepancy between inhibition of *C. burnetii* growth upon direct addition of MES or CTC to bacterial cultures, and the lack thereof or enhanced replication in macrophages, raises several questions.

First, we do not know by which mechanism(s) ITA and its isomers inhibit *C. burnetii* growth in the ACCM-2 culture medium. Direct antibacterial activity of ITA has been demonstrated first against *M. tuberculosis* through inhibition of isocitrate lyase (ICL) that feeds the glyoxylate shunt (5) and is also the mechanism relevant for inhibition of *Brucella melitensis* (18). A metabolite of ITA, itaconyl-CoA inhibits methylmalonyl-CoA mutase of *M. tuberculosis*, thereby blocking propionate-dependent growth (28). *C. burnetii* does not express ICL and lacks enzymes of the glyoxylate pathway, suggesting that ITA’s inhibitory effect on *C. burnetii* is likely caused by another mechanism. ITA possesses a marked bactericidal effect on *C. burnetii* when used at a concentration of 5 mM, evidenced at the ultrastructural level by shrinkage of the bacterial cell and intracellular calcifications indicative of bacterial cell death (19). Both MES and CTC efficiently blocked *C. burnetii* growth, especially at the 5 mM concentration. However, compared to ITA, the bactericidal effect was reduced in the case of MES and not significant for CTC. Given that CTC acts bacteriostatic, while ITA and, to some extent, also MES are bactericidal, it is unclear whether all three isomers act by a common mechanism on *C. burnetii*. Future metabolic flux analysis should reveal whether ITA and its isomers inhibit central metabolic pathways in *C. burnetii* (29).

The second question concerns the lack of inhibition of *C. burnetii* growth in macrophages by MES and CTC. Our GC-MS data demonstrate that intracellular MES levels are comparable to those of ITA following exogenous addition to resting and infected macrophages, respectively. While ITA effectively reduces *C. burnetii* replication in infected macrophages, the failure of MES to do so despite similar intracellular levels may be due to a difference in the concentration of both isomers in the CCV. The intracellular trafficking of ITA (and its isomers) is only beginning to be elucidated. In *Salmonella*-infected macrophages, ITA produced in the mitochondria is transported to the *Salmonella*-containing vacuole in a Rab32-dependent process that involves the kinase LRRK2 (6, 30). Whether exogenously added ITA, MES and CTC are also dependent on this transport pathway in *C. burnetii*-infected macrophages remains to be tested. In addition, it is yet unknown how ITA and its isomers may be transported across the membrane of the CCV to reach the bacteria, and it is possible that differences in this process are responsible for the observed lack of control of intracellular *C. burnetii* by MES. In contrast, the intracellular concentration of CTC achieved after exogenous supplementation is considerably lower than the concentrations observed for ITA and MES, potentially explaining the lack of anti-*Coxiella* activity in *Acod1^-/-^* macrophages.

In fact, CTC strongly enhanced replication of *C. burnetii* in *Acod1^+/-^* macrophages, which can be attributed to a significant reduction in the generation of endogenous ITA (**Figure 4A**), consistent with the inhibition of ACOD1 enzymatic activity by CTC. Interestingly, in *Acod1^-/-^* macrophages, CTC exerted a weaker growth-promoting effect on *C. burnetii*, indicating that this isomer may impair the anti-microbial capacity of macrophages by additional mechanisms, which may be linked to its high electrophilicity, e.g. induction of NRF2-dependent gene expression or inhibition of ROS production (25).

The reduction of ITA production in MES-treated *C. burnetii*-infected macrophages (**Figure 4A**) was surprising, because MES had not been reported to inhibit ACOD1 enzymatic activity and has less structural similarity to its substrate cis-aconitate (25). We considered it possible that MES may also inhibit ACOD1 at the concentration of 2.5 mM used here, but the results of ACOD1 enzyme activity assays in the presence of MES excluded this possibility. We also excluded reduced expression of *Acod1* in the presence of MES as mechanism for the reduction in intracellular ITA levels. Another possible mechanism underlying this effect may be the inhibition of uptake of extracellular ITA that we observed here after addition of exogenous ITA to *Acod1^-/-^* macrophages (**Figure 5C**) and that may also block uptake of secreted endogenous ITA from the culture supernatant.

This reduced uptake of exogenous ITA into macrophages co-treated with MES, but not with CTC (**Figure 5C**), indicates that ITA and MES may be transported into macrophages by the same mechanism and that MES competes with ITA. This notion of a shared uptake mechanism for ITA and MES is also supported by our observation that the uptake of exogenous ITA and MES is strongly enhanced in macrophages after infection with *C. burnetii* (**Figure 4B, E**), whereas the intracellular concentration of CTC in supplemented macrophages is not changed upon infection (**Figure 4F**). Thus, transport of extracellular ITA and MES into macrophages appears to be mediated by the same receptor/mechanism, which is up-regulated in infected macrophages. In contrast, CTC is likely taken up *via* a different, constitutively active transporter/mechanism.

Although exogenous MES and CTC are taken up by BMM and are readily found in our GC-MS assay, endogenous production of both isomers by resting or *C. burnetii*-infected macrophages was not observed in this study. In the case of MES, the reportedly low levels of MES compared to ITA in activated macrophages (less than 10%) (25, 26), coupled with the limited sensitivity of our detection method (LLQQ 0.098 µM in the method used Chen et al. (27) vs. 0.888 µM for MES in our GC-MS), likely explains our failure to find endogenous MES. The source of endogenous CTC is enigmatic. It does not appear to be produced by myeloid cells, at least not in the human THP-1 macrophage cell line (25). Therefore, while both MES and CTC are interesting molecules for potentially modulating ACOD1-ITA dependent biological response, the contribution of endogenously produced MES and CTC in macrophages may be limited.

Treatment of infected BMM with the isomers reduced the production of cytokines but did not abrogate it, consistent with previous publications on MES and CTC (25, 26). As no strong difference in macrophage activation was found between the isomer treatments that could be correlated to the effects on bacterial numbers, we conclude that the differential effects on bacterial burden are likely not caused by inhibition of cytokine production. Of note, there was no significant difference in the amount of cytokines measured in the supernatants of *Acod1^+/-^* and *Acod1^-/-^* BMM after *C. burnetii* infection (**Figure 3**), which may be surprising given the literature on the regulatory effect of endogenous ITA on several cytokines (13–15) and our previous demonstration of increased cytokine expression in tissues after *C. burnetii* infection in *Acod1^-/-^* mice (19). That endogenous ITA did not strongly affect the output of cytokines by infected BMM here is likely due to differences in timing (24 hours after the end of the BMM infection period vs. 7 days *in vivo*) and concentration of ITA (higher with exogenous ITA).

## Concluding remarks

Our comparative study revealed that all isomers inhibit cytokine production by *Coxiella*-infected macrophages and suppress replication of *C. burnetii* in axenic culture, but only ITA can inhibit intracellular growth of *C. burnetii*. The reasons for this divergence involve inhibition of ACOD1 enzyme activity in case of CTC. As all three isomers are candidates for therapeutic intervention in inflammatory processes, the differences we observed here in enhanced or thwarted control of intracellularly bacterial replication are important because they may interfere with protective host responses to acute infection or promote reactivation of latent infections. Specifically, it will be interesting to determine whether CTC increases bacterial burden in our *C. burnetii* infection model in mice via inhibition of ACOD1 activity.

The increased uptake of ITA (and MES) into infected as compared to resting macrophages suggests that endogenously produced extracellular ITA does not affect all cells in the microenvironment equally. Instead, by a regulated uptake mechanism, ITA may be preferentially delivered to cells that are activated, e.g. by intracellular infection or PRR-ligands in the tissue, and are more sensitive to ITA due to increased expression or activity of transporters mediating the uptake. The future identification of the molecules involved in the uptake and intracellular transport of ITA and its isomers will be important to provide more insight into the mechanistic regulation of ACOD1-ITA effects in inflammation and host defense.

## Materials and methods

### Reagents

Itaconic acid (ITA) and its isomers mesaconic acid (MES) and citraconic acid (CTC) were purchased from Sigma-Aldrich (Deisenhofen,Germany) (ITA, I29204-100G; MES, 131040-10G; CTC, C82604-5G). Stock solutions were prepared in PBS.

### Mice

The *Acod1^+/-^* and *Acod1^-/-^* mice utilized in this study were purchased from Jackson Laboratory (C57BL/6NJ-*Acod1^em1(IMPC)J^/*J) and bred at the *Präklinische Experimentelle Tierzentrum* of the University Hospital Erlangen (PETZ). Mice were humanely sacrificed to obtain bone marrow cells according to §4 of the German Animal Protection Law (Protocol Number TS-40/2021). Both male and female mice were used and groups were matched for sex as closely as possible.

### Culture of *Coxiella burnetii*

We used the avirulent Nine Mile phase II (NMII) strain of *C. burnetii* in this study. An isolate of the NMII strain clone 4 (NMII, RSA493) was generously provided by Matteo Bonazzi (Institut de Recherche en Infectiologie de Montpellier, Montpellier, France). One aliquot of purified NMII was propagated in a 75-cm^2^ tissue culture flask containing 30 mL of acidified citrate cysteine medium (ACCM-2, 4700-003, Sunrise Science Products, San Diego, CA, USA) at 37°C in a humidified atmosphere of 5% CO_2_ and 2.5% O_2_ (Omsland et al., 2009). After five days of culture, bacteria were pelleted for 15 min at 4,500 x g, resuspended in 1 mL phosphate buffered saline (PBS), and quantified by optical density at 600 nm (OD_600_), where an OD_600_ of 1 equals 1 x 10^9^ C*. burnetii* per mL. Bacteria were diluted in cell culture media without antibiotics and kept on ice until use for infection to macrophages.

### Treatment of *C. burnetii* with ITA and its isomers

Five mL of ACCM-2 media were inoculated with *C. burnetii* at a concentration of 10^6^ *Coxiella*/mL and propagated at 37°C, 5% CO_2_ and 2.5% O_2_. From the beginning of culture bacteria were treated with ITA, MES and CTC, respectively, for the indicated time periods. PBS was added as control to the bacterial culture to determine the optical density. For the determination of colony forming units (CFU), ITA, MES, CTC, and PBS were added on day 5 of bacterial culture and on day 7 bacterial culture was centrifuged at 4,500 x g for 15 min and the pellet was resuspended in 200 µL ACCM-D. Ten µL of serial dilutions were dropped on ACCM-D/0.3% agarose plates in triplicate. The plates were incubated for 2 weeks at 37°C, 5% CO_2_ and 2.5% O_2_. CFU were calculated according to the corresponding dilution factor. To measure bacterial growth by optical density, *C. burnetii* cultures were propagated as mentioned above. At the indicated time points, 100 µL were directly taken from the culture to determine the optical density at OD_600_.

### Bone marrow derived macrophages

Bone marrow cells were isolated from femurs and tibiae of mice and differentiated into macrophages in complete Dulbecco’s modified Eagle medium (cDMEM; DMEM [Life Technologies] plus 10% fetal bovine serum [FBS; Biochrom, Berlin, Germany], 50 μM β-mercaptoethanol, 1% penicillin and streptomycin, complemented with 10% L929 cell-conditioned medium (LCCM) as a source of macrophage colony-stimulating factor (M-CSF) in petri dishes at 37°C, 5% CO_2_ and 21% O_2_. After overnight culture, non-adherent bone marrow cells were counted and plated in Petri dishes at a density of 5 to 8 x10^6^ per 10-cm dish in cDMEM with 10% LCCM. Additional 5 ml of cDMEM with 10% LCCM were supplied per Petri dish on day 3 of culture. At day 6 adherent macrophages were harvested with Accutase (Sigma, Deisenhofen, Germany), washed and plated (1.5 x 10^5^ cells/well in a 96-well plate, 1 x 10^6^ cells/well in a 6-well plate) in cDMEM without antibiotics for infection the next day.

### Infection of macrophages with *Coxiella burnetii*

BMM were infected with *C. burnetii* at MOI 10 (based on OD_600nm_ measurement) and incubated at 37°C and 5% CO_2_. Four hours after infection, cells were washed with antibiotic-free cDMEM media to remove any extracellular bacteria and supplemented with itaconate and its isomers in cDMEM media without antibiotic. At the indicated time point supernatants were collected for cytokine measurement (24 hours) and the cells were lysed in PeqDirectLysis buffer (Peqlab/VWR 30-2010) with Proteinase K (PeqLab, Germany) for DNA preparation (for 4-hour and 96-hour time points).

### DNA isolation and quantification of *C. burnetii* burden via qPCR

BMM with PeqDirectLysis buffer plus proteinase K were incubated at 56°C overnight for lysis followed by an additional 45 minutes of incubation at 85°C to inactivate the proteinase K. QPCR was done from the BMM DNA samples. Bacterial load per cells was defined by the ratio of *C. burnetii* genomic copies to BMM genomic copies. To determine the genomic copies of *C. burnetii* we performed a TaqMan-based quantitative PCR for the insertion sequence IS1111 using 5’-CATCGTTCCCGGCAGTT-3’ and 5’-TAATCCCCAACAACACCTCCTTA-3’ as forward and reverse primer, and 6FAM-CGAATGTTGTCGAGGGACCAACCCAATAAA-BBQ as the internal fluorogenic probe (TibMolbiol, Berlin, Germany). Genome equivalents of *C. burnetii* were determined using a standard curve prepared from a known number of *C. burnetii* from *in vitro* culture. Genomic copies from host cells were quantified from the same DNA sample for murine albumin gene (exon 7) by using 5′-GGCAACAGACCTGACCAAAG-3′ and 5′-CAGCAACCAAGGAGAGCTTG-3′ as forward and reverse primer. Here we also determined the cell number using a standard curve from murine genome generated by serial dilution of DNA from mouse spleen cells. Finally, *C. burnetii* burden was calculated per cell by normalizing bacterial genome copy numbers to albumin copy numbers. An ABI Prism 7900HT sequence-detection system was used to perform the qPCR in 384-well optical plates. *C. burnetii* DNA was quantified by using 2x Roche FastStart Universal Master Mix (6 µL) together with 0.2 µM final concentration of each primer and an internal fluorogenic probe of 20 µM. Murine albumin gene was quantified by using 2x SYBRselect master mix together with 0.83 µM of each primer. For both qPCRs, 2 µL of isolated DNA (prediluted 1:4 in ultrapure H_2_O) was used as template in a final volume of 12 μL per reaction.

### GC-MS analysis

We measured the intracellular concentration of ITA, MES and CTC by GC-MS. On the day before infection, BMM were seeded (1 x 10^6^ cells/well) in 6-well plates in cDMEM without antibiotics. The next day, cells were infected with *C. burnetii* at MOI-10. Extracellular bacteria were washed away with cDMEM media four hours after infection, then ITA and its isomers (MES and CTC) as a single or combined treatment were added to the culture. 24 hours later, BMM were washed 4 times with PBS (1mL/well), with careful removal of PBS to ensure complete removal of extracellular ITA and isomers. The macrophages were harvested with ice-cold 80% methanol and the lysates were immediately frozen at -80°C until further analysis as recently described (31, 32). Briefly, for further analysis, the samples were thawed, vortexed and then centrifuged at 10,000 g at 4°C for 5 minutes. The extract was collected, and the remaining pellet was re-extracted twice with 200 μL of 80% methanol employing 15,000 g for the final centrifugation step. All extracts of a sample were combined and evaporated to dryness using an infrared vortex vacuum evaporator (CombiDancer, Hettich AG, Baech, Switzerland). The dried sample was reconstituted in 100 μL of pure water, transferred to a flat-bottom insert in a 1.5 mL glass vial and dried again for subsequent GC-MS analysis. The latter was performed using an Agilent GC model 6890 (Agilent, Palo Alto, USA) with an MSD model 5975 Inert XL and an MPS-2 Prepstation sample robot (Gerstel, Mühlheim, Germany) for automated methoximation and silylation as derivatization. An RXI-5MS column (30m × 0.25mm ID × 0.25 μm film thickness; Restek, Bad-Homburg, Germany) with a 2-m precolumn was used. The temperature program started at 50°C for 1 minute, then the column temperature was ramped up to 120°C at 5 K/min and then to 300°C at 8 K/min and, finally, held for 5 minutes. An injection volume of 1 μL and splitless injection at 280°C were used. During sample preparation, 10 µL of an internal standard solution containing ^13^C_5_-ITA (Toronto Research Chemicals/Biozol, Eching, Germany) at a concentration of 200 µM in pure water was added to the samples. For quantification, serial dilutions of a standard solution containing the three isomers were analyzed. Baseline separation of the three isomers was achieved (**Figure 4A**). To generate calibration curves, the peak areas (quantifier ion m/z 259.1) of ITA, CTC and MES were normalized with the peak area of the internal standard ^13^C_5_-ITA (quantifier ion m/z 264.1). LLOQs, calculated as concentration in the final derivatized sample (110 µL), were 0.444 µM for ITA and CTC, and 0.888 µM for MES. Metabolite concentrations were normalized to the total protein amount determined using the fluorescent dye SERVA Purple (Serva, Heidelberg, Germany) as described previously (Berger et al, 2021).

### *In vitro* ACOD1 assay

The inhibition of human and mouse ACOD1 by MES was tested in an *in vitro* assay using purified proteins as described (33), but using the buffer 50 mM MOPS, pH 7.5, 100 mM NaCl. 50 mM stocks of CTC and MES were prepared in water and neutralized with NaOH. The assay was performed with 3 mM, 5 mM and 10 mM MES or CTC, or without inhibitor, and produced ITA was measured by HPLC, as described (33).

### RNA isolation and *Acod1* mRNA analysis by qRT-PCR

RNA from BMM was isolated using TriReagent (Sigma-Aldrich, Deisenhofen, Germany) and cDNA was synthesized using High Capacity cDNA Reverse Transcription Kit (Applied Biosystems). Primers and probe for *Acod1* were chosen from the Universal Probe library (Roche). The fold change in gene expression was determined by the ΔΔCT method, using HPRT as housekeeping gene for calculation of ΔCT values and samples of naïve wild type or heterozygous mice as calibrators.

### ELISA

Cytokine concentrations were measured from cell culture supernatant collected at 24 hours post treatment. Cytokine concentrations in the supernatants were measured by DuoSet ELISA kits following the standard protocol of R&D systems.

### Statistical analysis

Biological replicates, numbers of mice used and total number of experiments are described in the figure legends. Statistical analysis was performed by GraphPad Prism software (version 9). Statistical significance was calculated using the Mann-Whitney test to compare two non-paired groups or the Friedman test for paired samples. Asterisks indicate different levels of significant p values (*<0.05, ** p<0.01, *** p<0.001, **** p<0.0001).

## Data availability statement

The raw data supporting the conclusions of this article will be made available by the authors, without undue reservation.

## Ethics statement

The studies were conducted in accordance with the local legislation and institutional requirements. Mice were humanely sacrificed to obtain bone marrow cells according to §4 of the German Animal Protection Law (Protocol Number TS-40/2021).

## Author contributions

MS: Writing – review & editing, Writing – original draft, Visualization, Investigation, Formal analysis. FK: Writing – review & editing, Methodology, Investigation, Formal analysis. MO: Writing – review & editing, Investigation. MZ: Investigation, Formal analysis, Writing – review & editing. KB: Formal analysis, Writing – review & editing. PO: Writing – review & editing. AL: Writing – review & editing, Supervision, Funding acquisition, Formal analysis. KD: Writing – review & editing, Supervision, Funding acquisition, Formal analysis. RL: Writing – review & editing, Writing – original draft, Visualization, Supervision, Project administration, Funding acquisition, Formal analysis, Conceptualization.

## References

[1] Degrandi D Hoffmann R Beuter-Gunia C Pfeffer K. The proinflammatory cytokine-induced IRG1 protein associates with mitochondria. J Interferon Cytokine Res. (2009) 29:55–67. doi: 10.1089/jir.2008.0013 19014335

[2] Bomfim CCB Fisher L Amaral EP Mittereder L McCann K Correa AAS. Mycobacterium tuberculosis induces irg1 in murine macrophages by a pathway involving both TLR-2 and STING/IFNAR signaling and requiring bacterial phagocytosis. Front Cell infection Microbiol. (2022) 12:862582. doi: 10.3389/fcimb.2022.862582 PMC9109611 35586249

[3] Li Y Zhang P Wang C Han C Meng J Liu X. Immune responsive gene 1 (IRG1) promotes endotoxin tolerance by increasing A20 expression in macrophages through reactive oxygen species. J Biol Chem. (2013) 288:16225–34. doi: 10.1074/jbc.M113.454538 PMC3675562 23609450

[4] Naujoks J Tabeling C Dill BD Hoffmann C Brown AS Kunze M. IFNs modify the proteome of legionella-containing vacuoles and restrict infection *via* IRG1-derived itaconic acid. PloS Pathog. (2016) 12:e1005408. doi: 10.1371/journal.ppat.1005408 26829557 PMC4734697

[5] Michelucci A Cordes T Ghelfi J Pailot A Reiling N Goldmann O. Immune-responsive gene 1 protein links metabolism to immunity by catalyzing itaconic acid production. Proc Natl Acad Sci U.S.A. (2013) 110:7820–5. doi: 10.1073/pnas.1218599110 PMC3651434 23610393

[6] Chen M Sun H Boot M Shao L Chang SJ Wang W. Itaconate is an effector of a Rab GTPase cell-autonomous host defense pathway against Salmonella. Science. (2020) 369:450–5. doi: 10.1126/science.aaz1333 PMC8020367 32703879

[7] Cordes T Wallace M Michelucci A Divakaruni AS Sapcariu SC Sousa C. Immunoresponsive gene 1 and itaconate inhibit succinate dehydrogenase to modulate intracellular succinate levels. J Biol Chem. (2016) 291:14274–84. doi: 10.1074/jbc.M115.685792 PMC4933182 27189937

[8] Sun P Zhang Z Wang B Liu C Chen C Liu P. A genetically encoded fluorescent biosensor for detecting itaconate with subcellular resolution in living macrophages. Nat Commun. (2022) 13 6562. doi: 10.1038/s41467-022-34306-5 36333306 PMC9636186

[9] O'Neill LAJ Artyomov MN. Itaconate: the poster child of metabolic reprogramming in macrophage function. Nat Rev Immunol. (2019) 19:273–81. doi: 10.1038/s41577-019-0128-5 30705422

[10] Day EA O'Neill LAJ. Protein targeting by the itaconate family in immunity and inflammation. Biochem J. (2022) 479:2499–510. doi: 10.1042/BCJ20220364 36546613

[11] Lang R Siddique M. Control of immune cell signaling by the immuno-metabolite itaconate. Front Immunol. (2024) 15. doi: 10.3389/fimmu.2024.1352165 PMC10938597 38487538

[12] Bambouskova M Gorvel L Lampropoulou V Sergushichev A Loginicheva E Johnson K. Electrophilic properties of itaconate and derivatives regulate the IkappaBzeta-ATF3 inflammatory axis. Nature. (2018) 556:501–4. doi: 10.1038/s41586-018-0052-z PMC6037913 29670287

[13] Lampropoulou V Sergushichev A Bambouskova M Nair S Vincent EE Loginicheva E. Itaconate links inhibition of succinate dehydrogenase with macrophage metabolic remodeling and regulation of inflammation. Cell Metab. (2016) 24:158–66. doi: 10.1016/j.cmet.2016.06.004 PMC5108454 27374498

[14] Mills EL Ryan DG Prag HA Dikovskaya D Menon D Zaslona Z. Itaconate is an anti-inflammatory metabolite that activates Nrf2 *via* alkylation of KEAP1. Nature. (2018) 556:113–7. doi: 10.1038/nature25986 PMC6047741 29590092

[15] Hooftman A O'Neill LAJ. The immunomodulatory potential of the metabolite itaconate. Trends Immunol. (2019) 40:687–98. doi: 10.1016/j.it.2019.05.007 31178405

[16] Gidon A Louet C Rost LM Bruheim P Flo TH. The Tumor Necrosis Factor Alpha and Interleukin 6 Auto-paracrine Signaling Loop Controls Mycobacterium avium Infection *via* Induction of IRF1/IRG1 in Human Primary Macrophages. mBio. (2021) 12:e0212121. doi: 10.1128/mBio.02121-21 34607464 PMC8546851

[17] Jessop F Buntyn R Schwarz B Wehrly T Scott D Bosio CM. Interferon gamma reprograms host mitochondrial metabolism through inhibition of complex II to control intracellular bacterial replication. Infect Immun. (2020) 88. doi: 10.1128/IAI.00744-19 PMC6977132 31740527

[18] Demars A Vitali A Comein A Carlier E Azouz A Goriely S. Aconitate decarboxylase 1 participates in the control of pulmonary Brucella infection in mice. PloS Pathog. (2021) 17:e1009887. doi: 10.1371/journal.ppat.1009887 34525130 PMC8443048

[19] Kohl L Siddique M Bodendorfer B Berger R Preikschat A Daniel C. Macrophages inhibit Coxiella burnetii by the ACOD1-itaconate pathway for containment of Q fever. EMBO Mol Med. (2023) 15:e15931. doi: 10.15252/emmm.202215931 36479617 PMC9906395

[20] Boyer MA Fischer NL Shin S. TNF and type I IFN induction of the IRG1-itaconate pathway restricts Coxiella burnetii replication within mouse macrophages. bioRxiv. (2023). doi: 10.1101/2023.07.07.548079

[21] Swain A Bambouskova M Kim H Andhey PS Duncan D Auclair K. Comparative evaluation of itaconate and its derivatives reveals divergent inflammasome and type I interferon regulation in macrophages. Nat Metab. (2020) 2:594–602. doi: 10.1038/s42255-020-0210-0 32694786 PMC7378276

[22] Chen LL Morcelle C Cheng ZL Chen X Xu Y Gao Y. Itaconate inhibits TET DNA dioxygenases to dampen inflammatory responses. Nat Cell Biol. (2022) 24:353–63. doi: 10.1038/s41556-022-00853-8 PMC9305987 35256775

[23] Zeng YR Song JB Wang D Huang ZX Zhang C Sun YP. The immunometabolite itaconate stimulates OXGR1 to promote mucociliary clearance during the pulmonary innate immune response. J Clin Invest. (2023) 133. doi: 10.1172/JCI160463 PMC10014103 36919698

[24] Chen C Zhang Z Liu C Sun P Liu P Li X. ABCG2 is an itaconate exporter that limits antibacterial innate immunity by alleviating TFEB-dependent lysosomal biogenesis. Cell Metab. (2024) 36:498–510. doi: 10.1016/j.cmet.2023.12.015 38181789

[25] Chen F Elgaher WAM Winterhoff M Bussow K Waqas FH Graner E. Citraconate inhibits ACOD1 (IRG1) catalysis, reduces interferon responses and oxidative stress, and modulates inflammation and cell metabolism. Nat Metab. (2022) 4:534–46. doi: 10.1038/s42255-022-00577-x PMC9170585 35655026

[26] He W Henne A Lauterbach M Geissmar E Nikolka F Kho C. Mesaconate is synthesized from itaconate and exerts immunomodulatory effects in macrophages. Nat Metab. (2022) 4:524–33. doi: 10.1038/s42255-022-00565-1 PMC9744384 35655024

[27] Winterhoff M Chen F Sahini N Ebensen T Kuhn M Kaever V. Establishment, validation, and initial application of a sensitive LC-MS/MS assay for quantification of the naturally occurring isomers itaconate, mesaconate, and citraconate. Metabolites. (2021) 11. doi: 10.3390/metabo11050270 PMC8146994 33925995

[28] Ruetz M Campanello GC Purchal M Shen H McDevitt L Gouda H. Itaconyl-CoA forms a stable biradical in methylmalonyl-CoA mutase and derails its activity and repair. Science. (2019) 366:589–93. doi: 10.1126/science.aay0934 PMC7070230 31672889

[29] Hauslein I Cantet F Reschke S Chen F Bonazzi M Eisenreich W. Multiple substrate usage of coxiella burnetii to feed a bipartite metabolic network. Front Cell infection Microbiol. (2017) 7:285. doi: 10.3389/fcimb.2017.00285 PMC5489692 28706879

[30] Lian H Park D Chen M Schueder F Lara-Tejero M Liu J. Parkinson's disease kinase LRRK2 coordinates a cell-intrinsic itaconate-dependent defence pathway against intracellular Salmonella. Nat Microbiol. (2023) 8:1880–95. doi: 10.1038/s41564-023-01459-y PMC10962312 37640963

[31] Hayek I Fischer F Schulze-Luehrmann J Dettmer K Sobotta K Schatz V. Limitation of TCA cycle intermediates represents an oxygen-independent nutritional antibacterial effector mechanism of macrophages. Cell Rep. (2019) 26:3502-3510 e3506. doi: 10.1016/j.celrep.2019.02.103 30917307

[32] Nanadikar MS Vergel Leon AM Guo J van Belle GJ Jatho A Philip ES. IDH3gamma functions as a redox switch regulating mitochondrial energy metabolism and contractility in the heart. Nat Commun. (2023) 14:2123. doi: 10.1038/s41467-023-37744-x 37055412 PMC10102218

[33] Chen F Yalcin I Zhao M Chen C Blankenfeldt W Pessler F. Amino acid positions near the active site determine the reduced activity of human ACOD1 compared to murine ACOD1. Sci Rep. (2023) 13:10360. doi: 10.1038/s41598-023-37373-w 37365251 PMC10293213

